# Media response to colon cancer campaigns in Switzerland 2005-2007: regional newspapers are the most reliable among the printed media

**DOI:** 10.1186/1756-0500-3-177

**Published:** 2010-06-24

**Authors:** Carine F Wang-Buholzer, Marta Lomazzi, Bettina Borisch

**Affiliations:** 1 Institute of Social and Preventive Medicine, University of Geneva, University Medical Centre, rue Michel Servet 1, 1211 Geneva 4, Switzerland

## Abstract

**Background:**

Health campaigns are frequently covered by printed media, but coverage is not homogeneous across different types of newspapers. Switzerland as a multilinguistic country with many newspapers offers a good field for study. A better understanding of how printed media report on national campaigns against colon cancer in the three main linguistic regions may help to improve future public health interventions. Therefore, we analyzed articles published between 2005 and 2007 during the campaigns "*Darmkrebs-nie*?" and "*Self-Care*" in the German, French and Italian regions of Switzerland.

**Findings:**

Some 65% of articles reporting on colon cancer were in German, 23% and 12% were in French and Italian respectively. During the campaign, topics linked to colon cancer were increasingly covered by the media. Regional newspapers (66%) reported significantly more about colon cancer and produced the most detailed articles.

Both gain- and loss-framed messages have been used by journalists, whereas the campaigns used merely gain-framed messages. Latin (French and Italian) newspapers mixed gain- and loss-framed messages in the same articles, while German articles mainly used a single frame throughout.

**Conclusions:**

Swiss-German papers reported more about the topic and the reporting was quantitatively and qualitatively more prominent in regional papers. The press followed the campaigns closely only during the period of campaigning, with high coverage. We propose to consider the regional press as an important vehicle of health information. Moreover, slight differences in framing can be observed between German and Latin articles.

## Background

Health is one of the main concerns in modern societies and messages regarding health occupy sizeable sections in the media. Health campaigns are a means of spreading such messages to the general public and mainly focus on preventive or protective measures. Despite their important coverage in the Swiss printed media, the effects of health campaigns have not been extensively studied in this context [1]. This is interesting because in Switzerland there is a high density of newspapers per inhabitant [2]. Moreover, a variety of daily papers are published in linguistically distinct communities. This makes printed media an important potential vehicle for public health messages. However, campaign designers in Switzerland have to overcome the linguistic barriers that split the country and the media into German-, French-and Italian-speaking communities. The media printed in German or French also include newspapers carrying information of national and international relevance (elite press) but their distribution does not exceed 5% [3]. A daily newspaper reflects the territorial identity and linguistic areas included in its distribution region [4]. Some daily regional papers in the German-speaking part of the country contain specific regional pages, whereas political, international, sports and cultural news are recaptured from more "general" newspapers [5].

The effect of health campaigns on behavioral changes in the population depends in part on the framing of the campaign message and the way it is relayed by journalists [6]. Health messages can be framed either in terms of potential gains or potential losses [7-9]. Mixed frame messages, including positive and negative frames in the same sentence, have been also described [9]. We hypothesize that there will be differences in the types of frames used in campaigns and articles as well as differences in frame usage in the different parts of Switzerland.

We focused on colon cancer campaigns since this medical condition represents a main public health concern in Switzerland with 4000 incident cases and 1600 deaths from colon cancer per year [10,11]. Over half of the new cases are diagnosed at advanced stages so that a public awareness of early symptoms and prevention seems necessary.

## Methods

We investigated how printed media responded to "*Darmkrebs-nie*?" [12] and "*Self-Care*" [13] colon cancer campaigns in Switzerland from 1^st ^September 2005 to 31 August 2007. The "*5 a day*" campaign (on-going during our inquiry) served as a baseline.

We used the ZMS/PMA Medienbeobachtung online archive of Swiss printed media to search for articles on colon cancer that mention the campaigns and 754 articles (corresponding to our research criteria) were kept for evaluation. The total numbers of articles of each speaking area were normalized to the relevant populations (4.64 Mio German; 1.49 Mio French; 0.47 Mio Italian).

We analyzed both the contents and the quality of the journal articles mentioning each campaign. First a coding exercise (for the items, see list) was done with three independent coders; then the coding consistency among them was tested followed by a second coding run. In addition, a content analysis was used as a formal approach to quantify and analyze the presence, meanings and relationships of the articles for media evaluation [14].

The texts were examined for the occurrence of the 11 most frequent items used by journalists in relation with the main topics of colon cancer prevention, diagnosis and treatment. The items were chosen for their pertinence to colon cancer and related health attitudes (see [Additional file 1: Supplemental Methods S1]). Moreover, we compared the proportion of articles written in the three main languages of Switzerland, versus the types of newspaper reporting on colon cancer, and observed the time-dependent variations in the number of reports. The number and kind of further information (websites, brochures) in articles was assessed.

The articles were also subjected to qualitative analysis: we investigated the frames used by the journalists to convey their message, in particular the presence of gain- or loss-framed messages.

The data were subjected to Student's T-test on SPSS (Statistical Package for the Social sciences). The *p*-value threshold used was 0.05.

## Results

Among the eligible articles, 666 were written in German (143.5 articles per million German-speakers), while 75 were written in French (50.3/Mio French-speakers) and 13 in Italian (27.6/Mio Italian-speakers). This analysis (Table 1) indicated a preponderance of articles in German (65%), compared to French (23%) and Italian (12%). Those articles were respectively found in 184 German (39.6/Mio German-speakers), 38 French (25.5/Mio French-speakers) and 10 Italian newspaper issues (21.3/Mio Italian-speakers) (Table 2). In general, the total number of newspapers per inhabitant was slightly higher in the French-speaking part of Switzerland (Table 3) [15]. Analyzing the distribution of the types of newspapers, it was found that most articles came from regional newspapers (66%), followed by 25% in magazines, 8.5% in elite press and 0.5% in tabloids (Table 4).

**Table 1:** Number of articles according to languages

Languages (Nb of speakers in Mio)	Nb of articles (per Mio speakers)	Total articles (%)
**German **(4.64)	666 (143.5)	65%
**French **(1.49)	75 (50.3)	23%
**Italian **(0.47)	13 (27.6)	12%

**Total**	754	100%

Selected articles were analyzed according to their language distribution and normalized to the number of specified language-speakers.

**Table 2:** Number of newspapers according to languages

Languages (Nb of speakers in Mio)	Nb of newspapers (per Mio speakers)
**German **(4.64)	184 (39.6)
**French **(1.49)	38 (25.5)
**Italian **(0.47)	10 (21.3)

**Total**	232

Selected newspapers-containing articles were analyzed according to their language distribution and normalized to the number of specified language-speakers.

**Table 3:** Total number of newspapers according to languages

Languages (Nb of speakers in Mio)	Nb of newspaper (per Mio speakers)
**German **(4.64)	7.9
**French **(1.49)	11.4
**Italian **(0.47)	8.5

Total Swiss newspapers were analyzed according to their language distribution and normalized to the number of specified language-speakers.

**Table 4:** Distribution of types of newspapers according to languages

Types of newspapers	German (4.64 Mio)	French (1.49 Mio)	Italian (0.47 Mio)	Total (%)
**Regional**	447 (96.3)	41 (27.5)	9 (19.1)	497 (66%)
**Magazines**	157 (33.8)	28 (18.8)	3 (6.4)	188 (25%)
**Elite press**	58 (12.5)	6 (4.0)	1 (2.12)	65 (8.5%)
**Tabloid**	4 (0.8)	0	0	4 (0.5%)

**Total**	666	75	13	754 (100%)

Distribution of types of newspapers in absolute numbers and normalized to the number of specified language-speakers.

**Figure 1:**
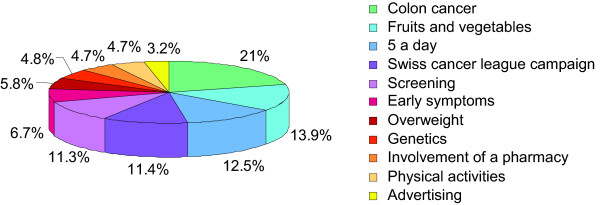
**Percentage of the 11 items quoted in selected articles**. Items reported: "Colon cancer", "Fruits and vegetables", "*5 a day*", "Swiss cancer league", "Screening", "Early symptoms", "Overweight", "Genetics", "Involvement of a pharmacy", "Physical activity" and "Advertising".

We then analyzed the themes preferentially covered by the media. The "Colon cancer" item was quoted in the 21% of the selected media (Figure 1). The item "Fruit and vegetables" came in second place (13.9%), followed by "*5 a day*" (12.5%), "Swiss cancer league" (11.4%), and "Screening" (11.3%). Other items such as "Early symptoms", "Overweight", "Genetics", "Involvement of a pharmacy", "Physical activity" and "Advertising" were mentioned less than 10%.

**Figure 2:**
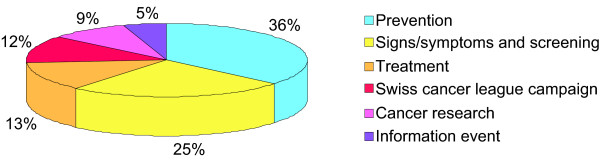
**Distribution of the six main themes used by the media**. "Prevention": nutrition/sports to protect against colon cancer; "Signs/symptoms and screening": all steps preceding diagnosis; "Treatment": care aspects; "Swiss cancer league campaign"; "Cancer research": scientific advances/new pharmacological treatment; "Information event": information demonstration in supermarkets/shops.

Among the eleven items, we chose six themes, representing the main health concerns conveyed by the campaigns. The three most cited ones were colon cancer prevention (36%), early signs/symptoms and screening (25%) and cancer treatment (13%; Figure 2).

**Figure 3:**
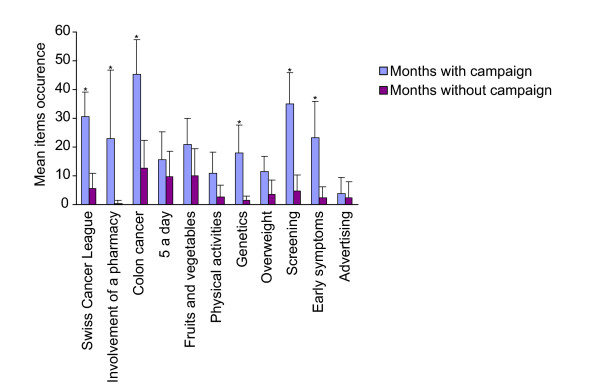
**Comparison of items mentioned by the printed media during and after the colon cancer campaigns**. Items occurrence during and after the Swiss Cancer League campaign (March 06 and March 07) and the PharmaSuisse campaign (August and September 06). Results were subjected to Student's T test. * signals that *p*-values are < 0.05.

When comparing the number of articles that mention the different items during the campaigns, we observed that "Swiss cancer league", "Involvement of a pharmacy", "Colon cancer", "Genetics", "Screening" and "Early symptoms" showed significant increase during the campaigns (Figure 3). But the reporting quickly declined after the campaign for the whole of Switzerland. No significant increase was described for "*5 a day*", "Fruit and vegetables", "Physical activity", "Overweight" and "Advertising" items.

The "*5 a day*" item showed independent variations during the two years examined. Outside campaigns, "Fruit and vegetables" and "*5 a day*" both had an average background level of one article every 2 months.

The occurrence of the eleven selected items mentioned per article and journal type was then investigated. Three groups were defined: minimal (1-3 items), in depth (4-7 items) and extensive reporting (8-11 items). The regional press contained the bulk of publications in all groups and was significantly more represented in the 8-11 items group than any other type of printed media (*p *= 0.046; Table 5).

**Table 5:** Number of items mentioned according to journal types

Journal types	Nb journal types	Mean nb of articles (S.D.)	T test	*p*-value
**Regional *v*s**	1	497.0	3.8	*p *= 0.063
**non-regional press**	3	85.7 (93.7)		
1 to 3 items	1	286.0	3.4	*p *= 0.075
	3	61.0 (56.6)		
4 to 7 items	1	181.0	3.9	*p *= 0.061
	3	21.7 (35.8)		
8 to 11 items	1	30.0	4.5	*p *= 0.046*
	3	3.0 (5.2)		
				

**Magazines *vs***	1	188.0	-0.002	*p *= 0.998
**non-magazines**	3	188.7 (168.8)		
1 to 3 items	1	116.0	-0.01	*p *= 0.993
	3	117.7 (148.9)		
4 to 7 items	1	63.0	0.02	*p *= 0.988
	3	61.0 (103.9)		
8 to 11 items	1	9.0	-0.1	*p *= 0.965
	3	10.0 (17.3)		
				

**Elite *vs***	1	65.0	-0.6	*p *= 0.625
**non-elite press**	3	229.7 (249.1)		
1 to 3 items	1	64.0	-0.4	*p *= 0.708
	3	135.0 (142.5)		
4 to 7 items	1	1.0	-0.8	*p *= 0.525
	3	81.7 (91.4)		
8 to 11 items	1	0	-0.7	*p *= 0.541
	3	13.0 (15.4)		
				

**Tabloid *vs***	1	4.0	-0.9	*p *= 0.439
**non-tabloids**	3	250.0 (222.6)		
1 to 3 items	1	3.0	-1.1	*p *= 0.374
	3	155.3 (116.1)		
4 to 7 items	1	1.0	-0.8	*p *= 0.525
	3	81.7 (91.4)		
8 to 11 items	1	0	-0.7	*p *= 0.541
	3	13.0 (15.4)		
				

The number of different items counted in each article (distributed in three groups: group I: 1-3 items, group II: 4-7 items and group III: 8-11 items) and corresponding journal type were listed. Results were submitted to Student's T test. * signals that *p*-values are < 0.05.

**Figure 4:**
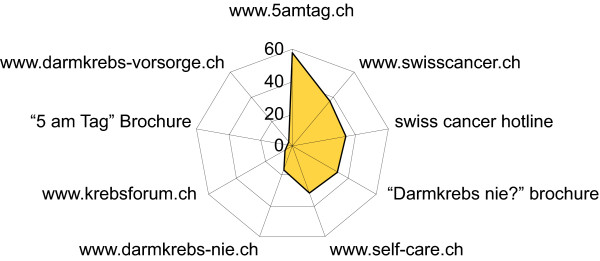
**Referral to relevant websites by printed media**. Occurrence of websites suggested by articles as sources for further information.

Some articles suggested websites as sources for further information (Figure 4). The 5amtag.ch website was the most often quoted by journalists (58 occurrences), followed by three references to the Swiss Cancer League: its website, a hotline and a specific brochure against colon cancer (36, 34 and 32 occurrences respectively). The link for the pharmaSuisse campaign website came in fifth, with 31 occurrences. Few announcements were given for other websites or brochures.

Finally, we focused on the framing of the main messages in the campaigns and articles. The campaigns adopted almost exclusively gain-framed messages (Table 6), whereas the articles reported the same contents using both gain- and loss-framed messages (Table 7). Several articles used both frames in one message ("mixed frame"). Of interest, many Latin articles mixed loss- and gain-framed message all along the article. Usually the initial message was a loss-framed one, while the last one was often gain-framed. In general there were more gain- than loss-framed messages. Many efforts were directed to children's education and prevention. The reporting in German was more uniform, indicating mainly gain-framed messages and focusing on themes such as the pleasure of eating healthy food (Table 7). Double negative constructions [16] were not used.

**Table 6:** Framing of the messages reported by the campaigns

Campaign	Original Text	Translated text
**"Darmkrebs-nie"**	*"Darmkrebs nie?"*	"Stop to colon cancer?"
	*"Ein gesunder Lebensstil* *verringert das Risiko einer* *Darmkrebserkrankung"*	"A healthy lifestyle reduces the risk of colon cancer"

**"Self-care"**	*"Darmkrebs-Vorsorge!* *Die Früherkennung ist für die Heilung wichtig. Lassen Sie sich in Ihrer Apotheke testen und beraten"*	"Colon cancer prevention The early detection is essential for the healing. Let your pharmacy make the test and advise you"

**"5 a day"**	*"5 Portionen Früchte und Gemüse pro Tag: und schon sind Sie dabei!"*	"5 portions of fruits and vegetables per day, off you go!"
	*"Fruchte und Gemüse machen fit und gesund"*	"Fruits and vegetables make you fit and healthy"
	*"Mit Fruchten und Gemüse gegen Übergewicht bei Kindern"*	"Fruits and vegetables against children overweight"
	*"Der Doktor empfiehlt: Fünfmal täglich"*	"The doctor suggests: 5 a day"

Examples of gain-framed messages reported by the campaigns "*Darmkrebs-nie*", "*Self-care*" and "*5 a day*". Almost no loss-gained messages have been detected.

**Table 7:** Gain- and loss-framed messages from selected articles

	Gain-framed message		Loss-framed message	
	
	Original Text	Translated text	Original Text	Translated text
**German Articles**	*"Nimm 5 am Tag...Genuss ohne Reue mit nachhaltiger Wirkung und wenig Aufwand"*	"5 a day...Benefit without regret with lasting effect and little expenses"	*"Warum tun nicht mal zwei Drittel der Schweizerinnen und Schweizer das Einfachste für ihre Gesundheit, indem sie täglich Früchte und Gemüse essen? Besonders vitamin-abstinent sind die Jungen"*	"Why two thirds of Swiss people do not do the simplest thing for their health, by eating daily fruits and vegetables? Mainly young people are vitamins abstinent"
	*"Kulinarischer Genuss im Namen der Gesundheit"*	"Culinary pleasure in the name of the health"	*"Darmkrebs kann jede und jeden treffen"*	"Colon cancer can touch everybody"
	*"Darmkrebs verhindern oder früh erkennen mit Hilfe Ihrer Apotheke"*	"Prevent and detect a colon cancer thanks to your pharmacy"		
	*"Darmkrebs - alle können etwas dagegen tun"*	"Colon cancer - everybody can do something against it"		

**French Articles**	*"Une vie saine limite les risques de cancer de l'intestin... Les facteurs bénéfiques les plus importants sont: pas de surcharge pondérale, une activité physique suffisante, une alimentation riche en fruits et légumes, peu de viande rouge et une consommation d'alcool modérée"*	"Healthy life can reduce colon cancer risk... It's mainly necessary to avoid overweight, have a good physical activity and eat a lot of fruit and vegetables while reducing red meat and alcohol consumption"	*"Les Suisses et les Suissesses boudent les fruits et les légumes...Seulement 18% des Suisses mangent les cinq portions recommandées quotidiennement"*	"Swiss people do not pay attention to fruit and vegetables... Only 18% of Swiss eat the 5 daily portions of fruits and vegetables recommended"
	*"Des gènes pourraient combattre les tumeurs"*	"Some genes can fight cancer"	*"Le cancer de colon fait des ravages en Valais"*	"Colon cancer is ravaging in Valais"
	*"La génétique prédictive est l'arme de demain"*	"Predictive genetic is the weapon of tomorrow"		

**Italian Articles**	*"...ma la tavola puo' anche essere una preziosa alleata se la dieta è ricca di frutta, verdura e cereali integrali"*	"...but well eating can be a precious friend if the diet is rich in fruits, vegetables and cereals"	*"Sono sempre piu' numerosi i ragazzi che soffrono di sovrappeso. I motivi risiedono nelle abitudini alimentari e nella mancanza di attività fisica"*	"More and more children suffer of obesity due to bad eating habits and lack of physical activity"
	*"Frutta e verdure combattono i tumori"*	"Fruits and vegetables fight against cancer"	*"Grassi e alcool aumentano il rischio di tumore"*	"Fat food and alcohol increase the risk of cancer..."

Example of different message framing in German, French and Italian articles reporting the campaigns.

## Discussion

Convincing public health messages are difficult to create and to communicate [14,15]. Health campaigns are an important means of health communication and often focus on the media. The Swiss printed media occupy an important position compared to neighboring countries and the density of newspapers is very high [15]. Therefore, health campaign planners in Switzerland should be knowledgeable about the interplay with the printed media. In Switzerland the presence of three main national languages adds complexity to the situation.

This study was undertaken in an attempt to analyze the printed media response to two colon cancer campaigns in Switzerland. Four main conclusions can be drawn from this work.

First, our results clearly showed a massive reporting of colon cancer in German newspapers (65%), which is not due to an overrepresentation of printed media in the region. There may be a cultural gap between the German and Latin parts of the country in receiving the same health message. The Swiss media are segmented in at least three distribution areas and thus Swiss citizens tend to address the same problems differently [5]. In spite of a large choice of media in various languages, three quarters of the population only consume written media in the official language in their area. It may well be that the reduced impact in Romandie (French-speaking) and Ticino (Italian-speaking) derives from the fact that the campaign was originally designed in German and then translated. Studies in interlinguistic equivalence in the medical field have indeed brought out the question of cultural identities in the translation of health vocabulary [17,18].

Second, the distribution of types of newspapers is interesting as two thirds of all selected articles were from regional newspapers. We discovered that most of them were local papers whose distribution was limited to small regions within Switzerland. Given the high local impact of this regional press, we offer for interpretation that these newspapers may be an efficient vehicle for public health information [19].

Third, we observed that the media response to both colon cancer campaigns was restricted to the duration of the campaign. Colon cancer is not adequately treated during 10 months per year. Such short term effects of campaigns have already been described [19,20].

The "*5 a day*" campaign had the same background level of reporting during the whole observation period. The efficiency of "*5 a day*" may be explained by its strong identity (an easy-to-remember slogan and a good logo).

Fourth, both loss- and gain-frames were used by the journalists, whereas the campaign itself was merely using gain-frames. According to prospect theory, even if the information within the messages is equivalent, the willingness to incur risk in order to obtain a desirable outcome or avoid an undesirable outcome changes depending on the message framing [20]. Several studies have shown that gain-framed messages, such as those issued by the campaigns, lead to a greater behavior shift then loss-framed ones. The articles show a nearly even distribution of both frames, especially in the Latin newspapers. However, since preferences in the mixed frame condition were virtually identical to the positive frame [21], we can consider media reported messages as gain-framed [22], thus suggesting that messages could be perceived by the reader as a motivation to change behavior.

Moreover, the influence of framing depends on the type of behavior which is promoted, which can be prevention or detection behavior. Among these two behaviors, a different degree of proximal risk is perceived, and prevention behaviors are usually considered less risky that detection ones [23]. For these reasons we hypothesize that "*Dramkrebs-nie*" and "*5 a day*" campaigns through their preventive attitude induce more behavior shift than "*Self-care*" which invites people to perform detection screening. The contents of the articles promote both behaviors, suggesting a prevention package linking a daily consumption of 5 portions of vegetables and fruit with regular physical activity and early detection by screening.

Changing health behaviors requires more than media communication and increasing health literacy. It largely depends on different enabling factors. First, self-motivation and personal skills are necessary to carry out successfully all of the tasks involved in changing behaviour. Second, human and structural health care resources are required to increase array of health information and health-related support services and extend the reach of health communication. Third community and environmental condition should be supportive of that change [24-26].

The next step would be to determine the impact of these messages on the population and ultimately their behavioral change. A pre- and post-campaign questionnaire study could be a first means of testing the uptake of the message as the campaign runs every September. However, the objective of this study was to analyze the quantitative and qualitative aspects of the reporting of the two campaigns; a further study should investigate the effects on awareness in the target population.

## Conclusions

We have shown that measurable differences exist between the German and the Latin part of Switzerland in reporting on colon cancer in the printed media, with the German press reporting most often.

Among the printed media, the regional press in Switzerland plays an important role in reporting health campaigns and this should be more exploited. The future of the local press is still under debate [27,28], but it seems to be at least a business success [28]. In this context health messages should more clearly target the regional printed media. Particular attention should be directed to the framing of the messages, taking advantage of the fact that positive framing can motivate people to adopt "positive" behaviors.

## Competing interests

The authors declare that they have no competing interests.

## Authors' contributions

CFWB collected the data and prepared them for publication. ML helped in writing and designing the article. BB conceived the study, created the contacts and led the study. All authors subsequently collaborated on finalizing the article.

## Supplementary Material

Additional file 1 **Supplemental Methods S1**. Eleven items coded (by two independent observers): "Colon cancer", "Swiss Cancer League", "Involvement of a pharmacy", "*5 a day*", "Genetics", "Screening", "Early symptoms", "Physical activities", "Fruits and vegetables", "Overweight" and "Advertising". These items have been chosen as they represent important key terms of the campaigns. For each article, several items could be registered. Click here for file
