# Supplementary material for: Media response to colon cancer campaigns in Switzerland 2005-2007: regional newspapers are the most reliable among the printed media
Source: BMC Res Notes. 2010 Jun 24;3:177. doi: 10.1186/1756-0500-3-177 (PMC2911466; doi:10.1186/1756-0500-3-177)
Supplement: Additional file 1 — Supplemental Methods S1. Eleven items coded (by two independent observers): "Colon cancer", "Swiss Cancer League", "Involvement of a pharmacy", "5 a day", "Genetics", "Screening", "Early symptoms", "Physical activities", "Fruits and vegetables", "Overweight" and "Advertising". These items have been chosen as they represent important key terms of the campaigns. For each article, several items could be registered. [file 1756-0500-3-177-S1.PDF]

Colon cancer:

Statements about colon cancer, including public health campaign or individual story.

Swiss cancer league:

Statements about the institution.

Involvement of a pharmacy:

Statements about pharmacies' involvement in colon cancer campaign, by either giving information to customers or proposing cheap and easy screening tests.

"5 a day":

Mentioning of the worldwide campaign "*5 a day*", which has encouraged for more than 15 years the consumption of a minimum of five fruits and vegetables a day.

Genetics:

Statements on the genetic factors predisposing for colon cancer.

Screening:

Statements on the screening methods for colon cancer, such as fecal occult blood test, or sigmoidoscopy and colonoscopy.

Early symptoms:

Statements on signs and symptoms of colon cancer: changes in bowel movements, feeling of bowel movement without any exoneration, rectal bleeding or blood in stool, cramping or steady abdominal pain, weakness and fatigue.

Physical activities:

Statements on the beneficial effects of physical activity against colon cancer.

Fruits and vegetables:

Statements on the beneficial effects of fruits and vegetables consumption as a protective measure against colon cancer.

Overweight:

Statements on overweight and obesity being risk factors for colon cancer.

Advertising:

Statement on advertisement being utilized to sell a particular health-promoting product (fruit juices, fruit preparations).
